# Hypokinesia upon Pallidal Deep Brain Stimulation of Dystonia: Support of a GABAergic Mechanism

**DOI:** 10.3389/fneur.2013.00198

**Published:** 2013-12-05

**Authors:** Florian Amtage, Thomas J. Feuerstein, Simone Meier, Thomas Prokop, Tobias Piroth, Marcus O. Pinsker

**Affiliations:** ^1^Department of Neurology, University Medical Center Freiburg, Freiburg, Germany; ^2^Section of Clinical Neuropharmacology, Department of Neurosurgery, University Medical Center Freiburg, Freiburg, Germany; ^3^Department of Stereotactic and Functional Neurosurgery, University Medical Center Freiburg, Freiburg, Germany

**Keywords:** deep brain stimulation, globus pallidus internus, subthalamic nucleus, idiopathic Parkinson’s disease, dystonia

## Abstract

In the past, many studies have documented the beneficial effects of deep brain stimulation (DBS) in the globus pallidus internus for treatment of primary segmental or generalized dystonia. Recently however, several reports focused on DBS-induced hypokinesia or freezing of gait (FOG) as a side effect in these patients. Here we report on two patients suffering from FOG after successful treatment of their dystonic movement disorder with pallidal high frequency stimulation (HFS). Several attempts to reduce the FOG resulted in worsening of the control of dystonia. In one patient levodopa treatment was initialized which was somewhat successful to relieve FOG. We discuss the possible mechanisms of hypokinetic side effects of pallidal DBS which can be explained by the hypothesis of selective GABA release as the mode of action of HFS. Pallidal HFS is also effective in treating idiopathic Parkinson’s disease as a hypokinetic disorder which at first sight seems to be a paradox. In our view, however, the GABAergic hypothesis can explain this and other clinical observations.

Deep brain stimulation (DBS) has become an established therapy for treatment of distinct movement disorders. Since its introduction in 1987 on patients with tremor (1), this therapeutic intervention has been used for a wide spectrum of diseases like idiopathic Parkinson’s disease (IPD), tremor, dystonia, and recently epilepsy. Moreover, studies and case series suggest efficacy in treatment of distinct psychiatric diseases. However, high frequency stimulation (HFS, i.e., stimulation above 100 Hz) of the internal globus pallidus (GPi) has been shown to be highly effective in segmental and generalized dystonia (2, 3), cervical dystonia (4, 5), and case series of tardive dystonia (6–9). Surprisingly, in the latest years, case reports and case series emerge, where pallidal HFS leads to hypokinesia and freezing of gait (FOG) in these patients (10–13). On the first sight this seems paradoxical, since pallidal HFS is also applied to sufficiently treat patients with hypokinetic IPD (14, 15). However, already in the first years of pallidal HFS for IPD, this controversy has been described, admittedly leaving the reason for that phenomenon unexplained (16).

So far, the mechanism of DBS is not known. Many hypotheses based on *in vitro* or animal studies do exist, explaining functioning of HFS/DBS via a “lesion effect” by reduced axonal output (17) or pronounced inhibitory input; for a detailed review see Feuerstein et al. (18) or McIntyre et al. (19). The GABAergic hypothesis (20) is one out of these assumptions, which, especially in its form of selective GABA release upon HFS (18), may explain the clinical findings and characteristics of subthalamic and pallidal HFS in IPD and dystonia, including their side effects.

This manuscript deals with the report of two patients who underwent stereotactic neurosurgery for pallidal DBS as a treatment for different types of dystonia. They both developed severe FOG in parallel to effective treatment of the dystonic movement disorder. We discuss these findings as supportive for the GABA-selective hypothesis of HFS and provide an explanation based on this hypothesis for the controversy mentioned above.

## Case 1

The first patient (caucasian female, 69 years) suffered from cervical dystonia with moderate torticollis to the right and laterocollis to the left including dystonic head tremor since 8 years. Wilson’s disease was ruled out by normal ceruloplasmin, serum-copper levels, and liver values. Treatments covered botulinumtoxin, trihexyphenidyl, tetrabenazine, and clonazepam without definite relief of the dystonic symptoms. Due to an intractable neck pain she developed reactive depression with sleep disturbances. Prior to planned stereotactic neurosurgery for DBS we conducted neuropsychological assessment which showed no cognitive decline. Psychosomatic and psychiatric consultation confirmed reactive depression, but represented no contraindication for operative treatment. DBS electrodes (DBS leads 3389; Medtronic Inc., Minneapolis, USA) were implanted in 04/2009 in local anesthesia. Post-interventional computed tomography (CT) and fusion with the preoperative magnet resonance imaging (MRI) showed correct locations of both DBS electrodes in the GPi without surgery-specific complications. The impulse generator (IPG) was implanted 1 day later (Activa PC, Medtronic Inc., Minneapolis, USA). The stimulation was introduced 3 days after implantation with an immediate beneficial effect on neck pain and a moderate effect for the dystonic movement including head tremor. Due to speech problems upon stimulation at the lowest contacts that we interpret as an effect on the internal capsule, we changed the stimulation parameters [contact 1/2(−) and 5/6(−) versus IPG(+): 2.0 V, 90 μs, 170 Hz]. Talairach stereotactic coordinates for stimulation contact on the left are *x* = −20 mm, *y* = 4 mm, and *z* = −4 mm, coordinates for stimulation contact on the right are *x* = 21 mm, *y* = 2 mm, and *z* = −2 mm in relation to the point in the middle of the line between the anterior and posterior commissure (mid-AC-PC-point; obtained by post-interventional computer tomography including stereotactic frame). FOG and limb hypokinesia were first recognized in 08/2010 after further increase of the stimulation parameters, accompanied by an optimal control of the head torsion (2.1 V, 150 μs, 180 Hz). The patient’s gait disturbances showed clear effects on levodopa therapy [UPDRS motor score (off/on): 13/7]; regrettably, no further ranking scales were applied at this time. We performed further investigations (FP-CIT-SPECT and sonography of the midbrain) which revealed no evidence of concomitant IPD. Furthermore, switch-off of the stimulation or reduction of the stimulation amplitude dramatically improved the patient’s gait, but induced certainly an increase of the dystonic movements. Unfortunately, it was not possible to identify stimulation parameters (e.g., bipolar stimulation, interleaved pulses, cyclic stimulation) with optimal control of the cervical dystonia in the absence hypokinetic side effects. Increasing the pulse width to heighten current density was also not helpful in this patient. We also applied stimulation the dorsal contacts which did not improve the FOG.

## Case 2

Our second patient (caucasian female, 63 years) suffered from a progressive generalized dystonia since childhood. The symptomatology consisted of severe retrocollis, torticollis to the right, opisthotonus of the spinal column and right accentuated limb dystonia with head and limb tremor. Molecular genetic analysis on DYT1 was negative, urine and serum probes for copper homeostasis showed no evidence for Wilson’s disease. MRI-scan revealed slight generalized brain atrophy. Tremor analysis of the right and left upper limbs revealed irregular dystonic tremor with a mean frequency of 4.6 and 3.5 Hz, respectively. Therapeutic attempts included levodopa, cervical botulinumtoxin injections, trihexyphenidyl, and baclofen, all without satisfactory effect. Thus, we performed bilateral pallidal DBS in 08/2008 (Kinetra, DBS leads 3389; Medtronic Inc., Minneapolis, USA). Post-interventional CT was merged with preoperative MRI and showed correct location of both DBS electrodes in the GPi without surgery-specific complications. During the course, the stimulation parameters were adapted showing the best improvement on mainly the distorted head, retrocollis, and tremor. Since monopolar stimulation lead to severe left-accentuated FOG, a bipolar setting to narrow the electrical field was chosen for the right GPi with the best compromise between good effect on dystonia and moderate distracting effect on gait [contact 1(−) versus IPG(+): 2.0 V, 240 μs, 130 Hz; bipolar setting contacts 5/6(−) versus 4/7(+): 2.3 V, 240 μs, 130 Hz]. More dorsal contacts were tentatively used, but did not lower the FOG. Talairach coordinates for stimulation contact on the left are *x* = −20 mm, *y* = 6 mm, *z* = −1 mm, coordinates for stimulation contact on the right are *x* = 21 mm, *y* = 5 mm, and *z* = −1 mm in relation mid-AC-PC-point (obtained by post-interventional computer tomography including stereotactic frame).

## Discussion

Two patients are presented here suffering from idiopathic dystonia who were treated by pallidal DBS with a satisfactory effect on the dystonic movement disorder. However, caused by HFS, a hypokinetic disorder of the legs has becoming manifest as FOG. This observation is cumulating in the past years, as patients with pallidal HFS present with hypokinesia during writing or walking (10–13). Levodopa responsiveness of these hypokinetic movement disturbances has not been described so far, but in our first case, the patient’s FOG improved after levodopa administration (see UPDRS). In our second case we did not try levodopa for individual reasons of the patient. Although the underlying pathophysiology of FOG induced by pallidal HFS cannot be comprehensively explained, we may suggest that it fits well into the GABA-selective hypothesis of HFS functioning.

The GABAergic hypothesis was first introduced by Dostrovsky et al. (20). In its form of HFS-induced selective GABA release (18), it is based on the assumption, that only GABAergic axons, but not other neuronal elements, respond to HFS and that the evoked action potentials propagate ortho- and antidromically along these axons. These action potentials only invade GABAergic terminals, based on experimental findings that HFS of human neocortical slices induces action potential-mediated exocytotic release of GABA, but not of glutamate (18, 21). The hypothesis of HFS-induced selective GABA release further proposes that HFS operates via opening of GABA_A_ autoreceptor-gated Cl^−^ channels of either terminal or somatodendritic location (18, 21). Since the corresponding Cl^−^ gradients in nerve endings favor a depolarizing Cl^−^ efflux (18, 21), terminal GABA_A_ autoreceptors act in a facilitatory manner, thereby enhancing GABAergic neurotransmission. Therefore, HFS of GABAergic axons leads to an enhanced GABA release from GABAergic terminals, resulting in an increased postsynaptic inhibition of the downstream nuclei.

The intention of the following discussion is to consider the clinical findings in patients with dystonia and Parkinson’s disease particularly with regard to the above mentioned hypothesis. It is not to review all arguments on that topic; for that we refer to the reviews of McIntyre et al. (19) and Feuerstein et al. (18). Our approach does not want to create the impression of neglecting other findings and to use a mechanistic and simplistic way to explain the mechanism of DBS or the basal ganglia network. We rather assume that the constellation of unique HFS parameters suggests a singular mechanism of action, corresponding to the principle of optimal simplicity in explaining observed effects.

**Figure 1:**
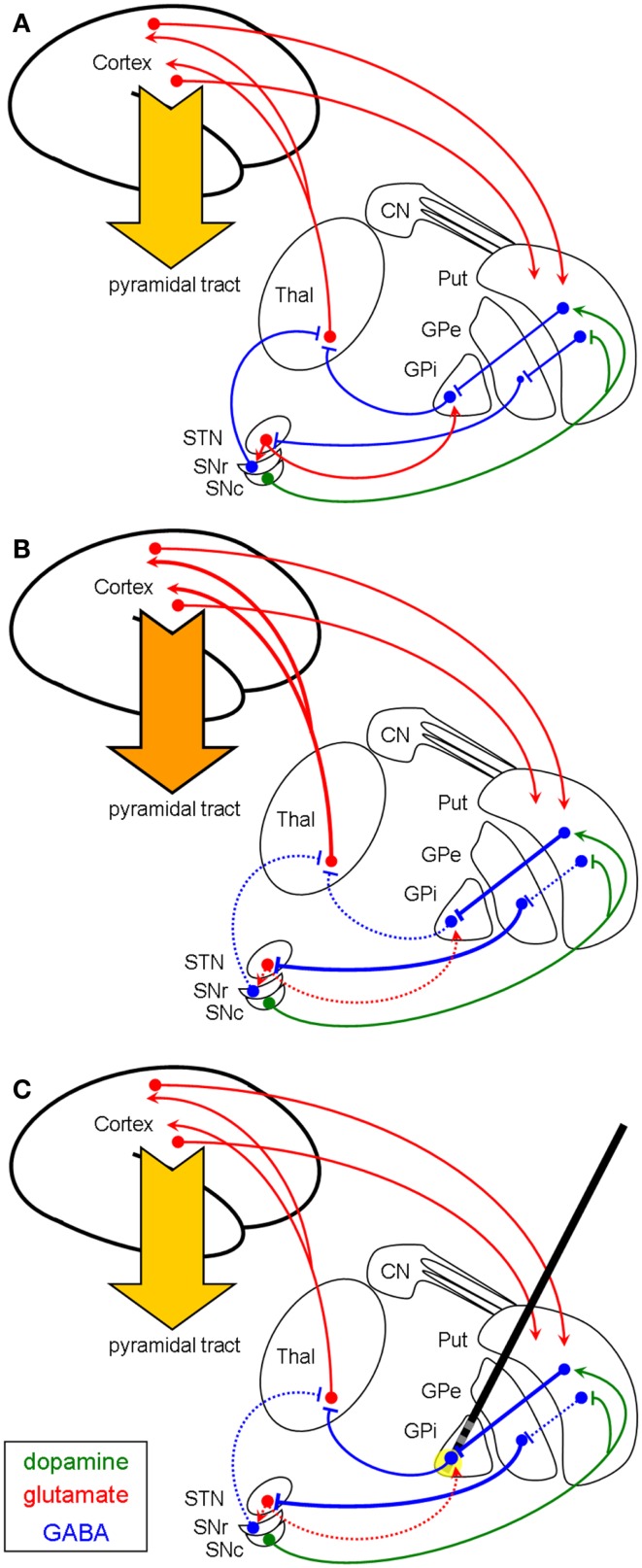
**Schematic illustration (coronar view) of the proposed basal ganglia network in a healthy condition (A) and in dystonia (B,C)**. In this context it is not yet established, if for the direct pathways the striato-pallidal efferents are overactive or if the indirect pathway (from Put to GPe) is reduced. **(B)** depicts both possibilities resulting in a reduced inhibition of the thalamus, leading to a hyperkinetic dystonic disorder. Following the GABAergic hypothesis, pallidal HFS [**(C)**, yellow circle] would result in a normalized thalamic inhibition via increased GABAergic outflow and therefore shows efficacy in reducing this hyperkinetic disorder (see also Discussion). Thal, thalamus; STN, subthalamic nucleus; SNr/c, substantia nigra pars reticularis/compacta; GPi/e, globus pallidus internus/externus; Put, putamen; CN, caudate nucleus; Arrowheads (green for dopamine, red for glutamate) reflect excitation; bars reflect inhibition (green for dopamine, blue for GABA). Pyramidal tract: dark orange arrow = hyperkinetic movement, light orange arrow = normokinetic movement.

In case of dystonia (Figure 1B), the GABAergic output of the GPi to the thalamus seems to be reduced. The underlying pathophysiology is not fully understood, but there is evidence for an exaggerated direct pathway (striato-pallidal fibers) with enhanced inhibition of the GPi. There also might be a reduced activity of the indirect pathway with less excitation of the GPi by subthalamic neurons [as reviewed in Hallett (22)]. Moreover, there is increasing evidence for an involvement of cerebello-thalamo-cortical circuits as well as disynaptic interconnections between basal ganglia and cerebellum in the pathophysiology of dystonia (23). Nevertheless, the thalamus is disinhibited and its glutamatergic efferents to the cortex provoke the hyperkinetic movement disorder. DBS for the treatment of dystonic movement disorders is usually targeting the ventro-medial parts of the GPi which contain the pallido-thalamic fibers. Applying the GABA-selective hypothesis on this pathophysiological concept, HFS of these GABAergic pallido-fugal axons would increase the GABAergic output onto the thalamus, resulting in a suppression of the hyperkinetic movements (Figure 1C).

**Figure 2:**
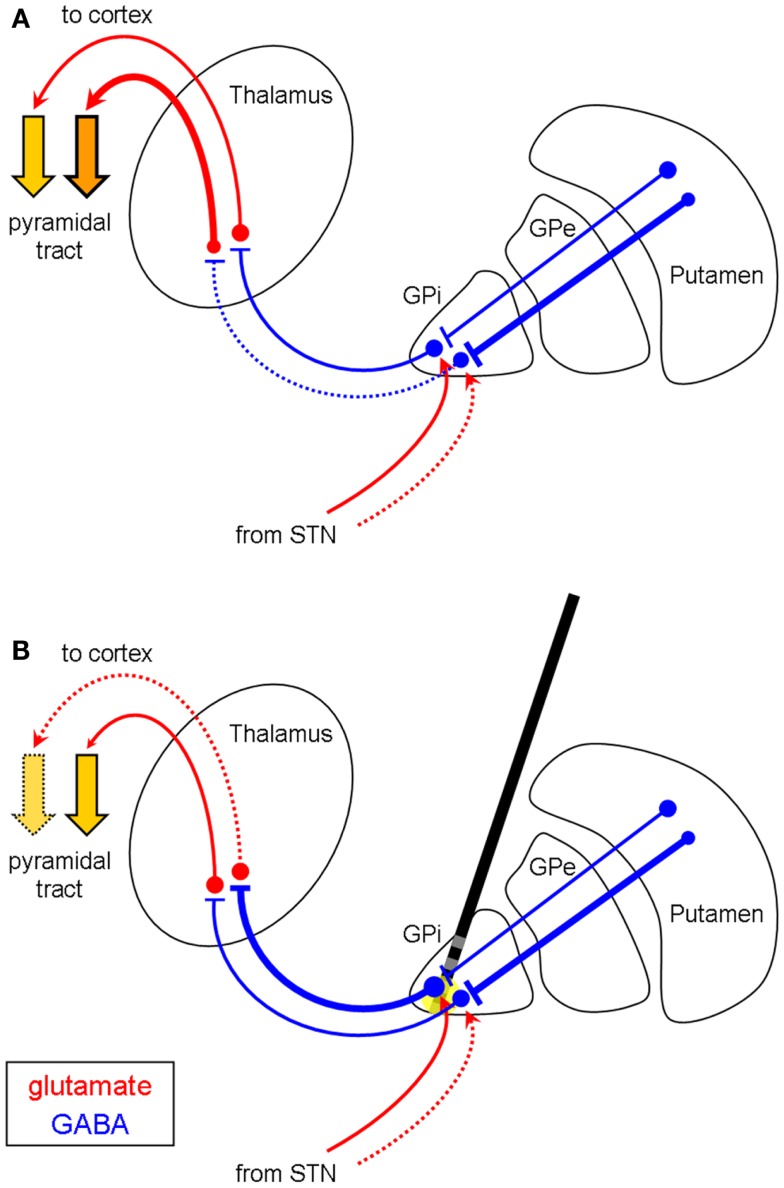
**Differential efficacy of pallidal HFS depending on the somatotopic distribution in focal/segmental dystonia (coronar view)**. **(A)** Disinhibition of the thalamus in the upper extremity (neurons in the ventro-lateral part of the GPi), but not in the lower limbs (medio-dorsal GPi), resulting in dystonia of the arms but not the legs. HFS of the medial GPi [**(B)**, yellow circle] increases the GABAergic outflow of the pallido-fugal fibers to the thalamus, which results in reduced hyperkinesia of the arms, but in hypokinesia (e.g., FOG) of the legs. Abbreviations and symbols are as in Figure 1. Pyramidal tract: dotted arrow = hypokinetic movement.

The somatotopic organization of the GPi reveals that neurons in the ventro-lateral and posterior portion are responsible for the upper extremity, whereas the representation of the lower limbs lies more medio-dorsally and anteriorly within the GPi (24–27). The area corresponding to the head and face is located medial and ventral to the region of the upper extremity (24). Hypokinetic side effects may occur when, depending on electrode localization, pallidal HFS leads to an enhanced GABAergic output of both the inhibited dystonic regions (e.g., in focal or segmental dystonia) and the non-affected neurons of the GPi (Figures 2A,B). Thus, the effect of HFS on the dystonic movement disorder may be experienced satisfactorily even though it induces hypokinesia, micrography, or FOG in many cases such as ours (Figure 2B). This might also explain the clinical observation that hyperkinetic movements such as dystonic tremor, dyskinesia, or kinetic movements within a dystonic syndrome respond more intensely to pallidal HFS than tonic movements, which correspond more likely to hypokinetic movements. Furthermore, the hypokinetic side effects of pallidal HFS are observations of the latest years, when focal or segmental dystonia were more frequently treated by implanted DBS electrodes. In generalized dystonia, however, i.e., when the whole body is covered by the pathophysiology, one would rarely expect these side effects. In our second patient, the left lower limb was least affected by the generalized dystonia, but, therefore, may have developed the greatest side effects of FOG as a hypokinetic phenomenon. Furthermore, levodopa was effective to treat FOG which was a side effect of pallidal stimulation in the first patient. We explain this by a levodopa-induced, D_1_ dopamine receptor-mediated rise in striato-pallidal inhibition of the non-affected part of the GPi, which then can be partly reversed, i.e., treated by the anti-hyperkinetic effect of pallidal DBS.

On the contrary, HFS of the GPi is effective for treatment of IPD, which is a typical hypokinetic disorder. Recently, a randomized-controlled study showed good clinical responses for both subthalamic or pallidal DBS in IPD with lack of a statistical difference between these groups (14). However, subthalamic stimulation (STN-HFS) is often preferred and judged to be superior to pallidal DBS, whereas the latter is frequently preserved for patients who developed slight psychosis or mild dementia. Without doubt, pallidal HFS is effective and increases quality of live in IPD patients, but it should be considered that dopaminergic medication cannot be significantly reduced after pallidal DBS as opposed to STN-HFS.

**Figure 3:**
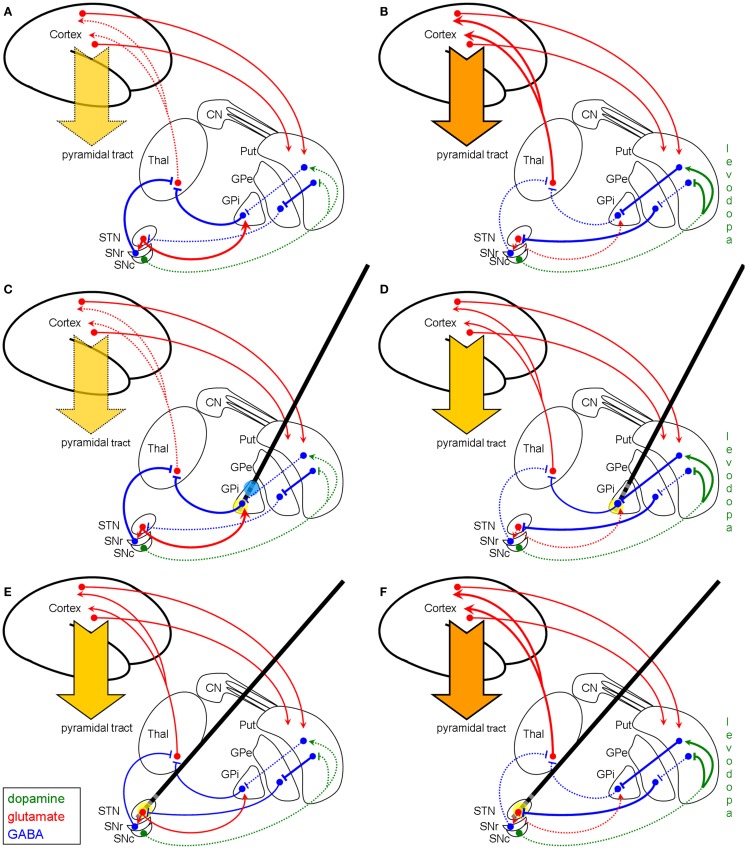
**Schematic model of the basal ganglia in IPD with and without modulation by HFS (coronar view)**. Left column **(A,C,E)**: basal ganglia model of hypokinetic IPD. Right column **(B,D,F)**: basal ganglia model of the hyperkinetic state in IPD after years of levodopa treatment. First row **(A,B)**: basal ganglia model without DBS modulation. Second row **(C,D)**: modulation of the network by pallidal HFS (yellow circle). Third row **(E,F)**: modulation of the basal ganglia network by STN-HFS (yellow circle). The GABAergic hypothesis illustrates why pallidal HFS is not effective in the hypokinetic state of IPD **(C)**, since the efferent GABAergic axons are maximally recruited, whereas STN-HFS shows good efficacy **(E)**. **(D)** displays that in the hyperkinetic state (i.e., levodopa-induced hyperkinesia) pallidal HFS is quite effective by producing an anti-hyperkinetic effect via enhanced GABAergic outflow on the thalamus, whereas STN-HFS *per se* is not effective to treat levodopa-induced dyskinesia **(F)**. The reason for this failure of STN-HFS is that the afferent fibers to STN are already maximally active. Note, however, that STN-HFS allows the reduction of the dosage of levodopa and thereby diminishes hyperkinesia. Blue circle in **(C)** indicates stimulation of the striato-pallidal fibers within the GPi as reconsidered in the discussion section. Abbreviations and symbols are as in Figures 1 and 2.

The basal ganglia network in the parkinsonian hypokinetic state is characterized by enhanced activity of the pallidal GABAergic efferents resulting in pronounced inhibition of the thalamus leading to hypokinesia (Figure 3A). Applying the GABA-selective hypothesis of HFS functioning on this pathologic IPD basal ganglia network, HFS of the fiber tract in the medio-ventral part of the GPi cannot further increase the GABAergic output to the thalamus and, thus, does not provoke anti-hypokinetic effects here, since the GABAergic transmission of the pallido-thalamic projection is already maximal (Figure 3C). Else in STN-HFS (Figure 3E): here an HFS-enhanced GABAergic input to the STN leads to a more physiological state of the STN itself and of its downstream nuclei. Moreover, pronounced STN-HFS even produces dyskinesia, as can be seen in some patients (28, 29). STN-HFS *per se* therefore exhibits anti-hypokinetic and pro-hyperkinetic effects. Thus, in a hyperkinetic state after levodopa medication, STN-HFS is not effective in alleviating dyskinesia (Figure 3F) as it is known from clinical practice. The reduction of dyskinesia upon subthalamic HFS is mainly due to a reduction of the dopaminergic medication. Nevertheless, we argue that the beneficial effect of HFS in the ventro-medial parts of the GPi in IPD may be founded on a symptomatic therapy of a hyperkinetic state owing to dopaminergic medication (Figure 3B), since the dopaminergic therapy often cannot be reduced after implantation. HFS of pallido-fugal axons enhances GABA release in the thalamus and thus causes an anti-hyperkinetic effect because of a normalized thalamo-cortical projection (Figure 3D). The result is an overall increased “on-time” during daytime and a better quality of life. However, producing a hyperkinetic state using an unchanged or even exaggerated dopaminergic medication will cause stabilization of the disorder due to the anti-hyperkinetic effect of pallidal HFS. This was already seen in the early years of pallidal DBS for IPD (16), where HFS on different locations within the GPi provoked distinct effects on bradykinesia and gait: stimulation of the postero-ventro-medial part of the GPi was found to worsen gait and bradykinesia (16), since pallidal HFS *per se* is pro-hypokinetic (see Figure 3C), but was found to reduce levodopa-induced dyskinesia by increasing GABA release in the thalamus (which is anti-hyperkinetic, see Figure 3D). In turn, HFS of the dorso-lateral part of the GPi was found to reduce hypokinesia and rigidity in IPD as a symptomatic effect of pallidal HFS (16). This also fits with the GABAergic hypothesis, since at this stimulation site (blue circle in Figure 3C) the incoming GABAergic fibers from the striatum are activated and thus enhance the inhibition by released GABA of the pallidal GABAergic projection neurons. Hence, the inhibition of the GPi is increased by HFS and, therefore, the thalamus gets disinhibited, resulting in an anti-hypokinetic effect at this simulation site (blue circle in Figure 3C).

The question arises why many dystonic patients do not have the above mentioned problem of hypokinesia as a side effect of pallidal stimulation. We suggest that this might be due to the above mentioned somatotopic organization of the GPi. When the stimulation is targeting mainly the somatotopic regions where dystonia is prominent, one could have a beneficial effect on dystonia without getting these side effects. Secondly, the number of patients suffering from this phenomenon might be underestimated: there are no systematic retrospective or even prospective evaluations of dystonia patients regarding hypokinesia or the ability to walk. We suppose that in a substantial portion of the patients with focal or segmental dystonia this side effect occurs, but is small in comparison to the benefit and therefore often neglected by the patient.

In conclusion, we used the hypothesis of HFS-induced selective GABA release to explain the hypokinetic side effects, i.e., FOG, of pallidal HFS for treatment of dystonia and the sensitivity of this side effect to levodopa treatment. Furthermore, this hypothesis suits well to describe the clinical observation that in dystonia hyperkinetic movements respond superiorly upon pallidal HFS in comparison to the response of tonic movements. The GABAergic theory beyond illustrates the mode of functioning of pallidal HFS in IPD, which is in line with the clinical experiences. In addition, the selective GABA release upon subthalamic HFS in IPD accounts for its symptomatic efficacy.

## Author Contributions

Florian Amtage – research project: conception, examination of the patients; manuscript: writing of the first draft.

Simone Meier – research project: examination of the patients; manuscript: review and critique.

Thomas J. Feuerstein – research project: conception; manuscript: review and critique.

Thomas Prokop – research project: examination of the patients; manuscript: review and critique.

Tobias Piroth – research project: examination of the patients; manuscript: review and critique.

Marcus O. Pinsker – research project: examination of the patients; manuscript: review and critique.

## Conflict of Interest Statement

The authors declare that the research was conducted in the absence of any commercial or financial relationships that could be construed as a potential conflict of interest.
